# Genetic and Pharmacologic Inhibition of Myostatin Restores Muscle Mass in a *Dynamin 2* ‐Related Centronuclear Myopathy Mouse Model

**DOI:** 10.1002/jcsm.70338

**Published:** 2026-09-11

**Authors:** Durieux Anne‐Cécile, Arnould David, Velarde Mathias, Allibert Valentine, Paret Chloé, Debelley Quentin, Kizilkilic Esra, Chambon Sidney, Girard Emmanuelle, Collin Louise, Gensel Alexandre, Castells Josiane, Pasternack Aja, Bowen Maximilien, Morel Baptiste, Hourdé Christophe, Laurent Schaeffer, Ritvos Olli, Bitoun Marc, Mounier Rémi, Freyssenet Damien

**Affiliations:** ^1^ Laboratoire Interuniversitaire de Biologie de la Motricité Université Jean Monnet Saint‐Etienne, Université Lyon 1, Université Savoie Mont‐Blanc Saint‐Etienne France; ^2^ Laboratoire Physiopathologie et Génétique du Neurone et du Muscle (PGNM), Institut NeuroMyoGene (INMG) Univ Lyon, Université Lyon 1 France; ^3^ Aniphy, SFR Santé Lyon‐Est Univ Lyon, Université Lyon1 Lyon France; ^4^ Department of Physiology, Faculty of Medicine University of Helsinki Helsinki Finland; ^5^ Centre de Biotechnologie Cellulaire Hospices Civils de Lyon Lyon France; ^6^ Inserm, Centre de Recherche en Myologie, Institut de Myologie Sorbonne Université Paris France

**Keywords:** Centronuclear myopathy, dynamin 2, muscle growth, myostatin

## Abstract

**Background:**

Autosomal dominant centronuclear myopathy (ADCNM), most commonly caused by mutations in the *dynamin 2* (*DNM2*) gene, is a rare congenital myopathy characterized by progressive muscle weakness and atrophy. Myostatin, a key negative regulator of skeletal muscle mass, has shown therapeutic potential in several models of neuromuscular diseases. We hypothesized that inhibiting myostatin could counteract muscle deconditioning in ADCNM and evaluated the therapeutic potential of both genetic and pharmacological myostatin inhibition strategies in a mouse model of ADCNM.

**Methods:**

Knockin‐*dnm2* ^R465W/+^ (KI) mice were first crossed with knockout‐*myostatin* mice (KO) to generate double mutant (KIKO) mice carrying both the R465W missense mutation and the *myostatin* gene deletion. In a second experiment, KI mice received intraperitoneal injections of a soluble activin type IIB receptor fused to IgG1 Fc fragment (sActRIIB‐Fc) at 5 mg kg^−1^ twice weekly starting at 4 weeks of age for 4 weeks. Muscle structure, molecular pathways and functional analysis were performed in *tibialis anterior* muscle.

**Results:**

MRI and immunohistochemical analyses showed that KI mice exhibited impaired postnatal muscle growth between 1 and 2 months of age, resulting in persistent muscle hypotrophy (−15%, *p* < 0.05). This defect was associated with a significant reduction in the number of satellite cells (−53%, *p* < 0.001 at 1 month and −68%, *p* < 0.01 at 2 months), the central accumulation of dense NADH‐TR staining in more than 20% of muscle fibres, an upregulation of atrophy‐related E3 ligases mRNA (*Trim63* + 50%, *p* < 0.001 *and* +87%, *p* < 0.001*; Fbxo32 + 39% p* < 0.05 *and* +95%, *p* < 0.001 at 1 and 2 months of age, respectively) and impaired autophagy. Genetic deletion of *myostatin* in KIKO mice fully restored muscle mass and normalized muscle function to wild‐type conditions. Pharmacological inhibition with sActRIIB‐Fc also robustly restored muscle mass to wild‐type values, primarily via activation of the Akt–mTOR pathway. However, this anabolic effect was transient, as muscle mass returned to baseline 5 weeks after treatment cessation. Importantly, despite increased muscle mass, sActRIIB‐Fc treatment did not improve muscle force and key pathological features, including defects in proteostasis, mitochondrial organization and excitation‐contraction coupling.

**Conclusions:**

These findings establish the proof of concept that myostatin inhibition counteracts skeletal muscle growth defect in ADCNM, which may be combined with other drugs to better address the multifactorial nature of muscle weakness in ADCNM.

## 1 Introduction

Centronuclear myopathies (CNM) are a group of rare congenital myopathies characterized by progressive muscle hypotrophy and weakness, with clinical severity ranging from fatal or severe neonatal forms to milder, late‐onset forms [1]. The histological signature of CNM is the abnormal central localization of nuclei in numerous muscle fibres, which gave its name to this group of myopathies [2]. Several genetic subtypes of CNM have been identified. The most severe form, X‐linked CNM (XLCNM), also known as myotubular myopathy, is caused by mutations in *MTM1*, encoding myotubularin [1]. Autosomal recessive CNM (ARCNM) most commonly results from gene mutations in *BIN1*, encoding amphiphysin 2, *RYR1*, encoding the ryanodine receptor 1, *SPEG*, encoding a myosin light chain kinase and *TTN*, encoding titin, a giant sarcomeric protein [1]. The autosomal dominant form (adCNM) is primarily caused by heterozygous mutations in the *DNM2* gene [3], which encodes dynamin 2, a GTPase involved in membrane fission, intracellular membrane trafficking and organelle morphology [4]. To date, approximately 40 heterozygous *DNM2* mutations have been associated with ADCNM [5], whereas only a single homozygous mutation has been reported, leading to a severe phenotype with premature death [6]. Mutations in *DNM2* account for approximately 15% of all CNM cases [7].

Myostatin, also known as growth and differentiation factor‐8, is a master negative regulator of skeletal muscle mass throughout development, postnatal growth [8] and adulthood [9, 10]. Myostatin signals through the activin type IIB receptor, which recruits a type I activin receptor and subsequently activates the SMAD2/3 signalling pathway [11]. This pathway regulates skeletal muscle mass by inhibiting the proliferation and differentiation of myogenic precursor cells [12], repressing the IGF1‐Akt–mTOR anabolic pathway [9], downregulating muscle‐specific gene expression [10] and promoting the expression of E3‐ubiquitin ligases involved in protein degradation [13]. Given its central role in regulating muscle mass, myostatin inhibition has shown promising effects in preventing muscle mass loss across several neuromuscular disease models [14], including XLCNM [15, 16].

While the development of genetic therapies for adcnm is crucial, addressing the loss of skeletal muscle mass and strength is also important to slow disease progression and improve patient's quality of life. In this study, we evaluated the therapeutic potential of both genetic and pharmacological myostatin inhibition in the KI‐*Dnm2* ^R465W/+^ mouse model of ADCNM [17]. Our molecular and functional analysis showed that both genetic deletion and pharmacological inhibition of myostatin corrected the skeletal muscle growth defect, with myostatin gene deletion also improving muscle function.

## 2 Methods

### 2.1 Animals

Knockin‐*Dnm2* ^R465W/+^ C57BL/6 J (KI mice) and knockout‐*myostatin* ^−/−^ C57BL/6 J (KO mice) mouse lines used in this study have been described previously [17, 18]. Animals were housed in filter‐top cages (2–4 mice per cage) with bedding and nesting enrichment in a temperature‐controlled room (20°C–21°C) under a 12‐h light/dark cycle, with *ad libitum* access to standard chow and water. To generate mice carrying both the heterozygous R465W *Dnm2* mutation and myostatin gene deletion (KIKO mice), KO female mice were crossed with KI male mice. Male *Dnm2* ^+/−^/*Myostatin* ^+/−^ offspring were then bred with KO females to obtain KIKO mice. KI, KO and KIKO male mice were identified by genotype analysis. All mice were used as they became available, allocated by genotype and paired with corresponding wild‐type (WT) littermates. Experiments were performed exclusively in male mice to reduce variability and ensure a consistent disease baseline. Although sex‐specific differences may exist, the mechanisms studied are expected to be relevant to both sexes. All procedures were conducted in accordance with the European Community guidelines and were approved by the Ministère de l'Enseignement Supérieur, de la Recherche, et de l'Espace and the local ethics committee for animal experimentation at Jean Monnet University.

### 2.2 Magnetic Resonance Imaging Analysis

MRI‐based longitudinal analysis of the cross‐sectional area of the lower limb leg muscles was performed at the mid‐belly (midpoint along the longitudinal axis) of the leg in WT and KI mice at 1, 2 and 8 months of age. Briefly, T1‐weighted images were acquired using a 7‐T Bruker Biospec MRI system (Bruker, Germany) with the following parameters: (TE/TR: 2.6 ms/25 ms; field of view: 3 cm × 1.5 cm × 1.9 cm; matrix size: 256 × 128 × 54; number of slices: 54; thickness: 352 μm; volume size: 3 cm × 1.5 cm × 1.9 cm; scan time: 35 min). AMIRA 3D visualization and analysis software (FEI, Oregon, USA) was then used for quantification.

### 2.3 Analysis of Motor Coordination and Grip Strength

Rotarod performance and grip strength of upper and lower limbs were determined in 1‐, 2‐ and 8‐month‐old WT, KI, KO and KIKO mice (Bioseb), as previously described [19].

### 2.4 Pharmacological Inhibition of Activin Type IIB Receptor

A soluble myostatin/activin A blocker (sActRIIB‐Fc) in the form of a recombinant fusion protein that contains the ectodomain of human activin type IIB receptor (ActRIIB) fused to a human IgG1‐Fc domain was produced as described earlier [20]. Briefly, the ectodomain of human ActRIIB and a human IgG1 Fc domain with a His6 tag were PCR‐amplified, subcloned into pGEM‐T vectors, sequenced, fused and inserted into the expression vector pEFIRES‐p. CHO cells were transfected with this vector, selected with puromycin and expanded first in DMEM and then in CD OptiCHO medium for large‐scale suspension culture. The protein was purified from culture supernatant using Ni^2+^‐affinity chromatography, dialysed and concentrated. Purity after IMAC purification was ~90% as determined by silver‐stained SDS‐PAGE. To determine the optimal dosage, sActRIIB‐Fc was first intraperitoneally injected twice a week for 4 weeks at 1, 5 and 10 mg kg^−1^. The optimal dosage (5 mg kg^−1^) was then used to treat 1‐month‐old KI male mice for 4 weeks. Mice were used for analysis at the end of the treatment (8‐week‐old mice) and 5 weeks after the cessation of the treatment (13‐week‐old mice). Age‐matched WT and KI control male mice received PBS as vehicle (hereafter referred to as WT‐PBS and KI‐PBS).

### 2.5 In Situ *Tibialis Anterior* Muscle Force Measurement

The distal tendon of the *tibialis anterior* muscle of isoflurane anaesthetized WT‐PBS, KI‐PBS and KI‐5 mg kg^−1^ mice was attached to the force‐position transducer apparatus (Aurora Scientific), while the knee was anchored and the electrodes positioned on the sciatic nerve. After determination of optimum muscle length, the force‐frequency relationship was determined (stimulation of 0.5 s at 25, 50, 75, 100 and 125 Hz at 2 mA spaced with 3 min recovery). To evaluate the contractile dynamics, the force (F)‐frequency (f) relationship was modelled by fitting the maximal force data obtained at each stimulation frequency with an increasing sigmoidal function. This relationship is defined by the following equation:

$$F\left(f\right)=\frac{F_{\max}}{1+e^{\frac{f_{50}-f}{\varphi }}},$$

where F(f) is the force produced at a given stimulation frequency f and φ is the slope parameter of the relation. The mathematical fit yields two independent parameters, F_max_, which represents the estimated asymptotic maximal force plateau, and f_50_, which corresponds to the stimulation frequency in Hz at which 50% of F_max_ is achieved. To account for differences in muscle size, the specific force (F_s_, in kPa), representing the maximal absolute force relative to the muscle physiological cross‐sectional area, was calculated as previously described [21] and adapted from recent muscle architecture data [22]. The absolute force (F in mN) was normalized to the muscle physiological cross‐sectional area in mm^2^ estimated using the muscle mass (m, in mg), muscle fibre optimal length (L_0_, in mm), muscle fibre length‐to‐L_0_ ratio (*R* = 0.47), pennation angle (*α* = 16.58°) and a mammalian muscle density (*ρ*) constant of 1.06 mg/mm^−3^. Normalized force (*Fs*) was then computed as:

$$F_{s}=\frac{F.\rho .R.L_{0}.\cos\left(\alpha \right)}{m}$$

### 2.6 Tissue Removal

Under deep anaesthesia and analgesia (intraperitoneal injection of ketamine 90 mg kg^−1^ and xylazine 10 mg kg^−1^), *extensor digitorum longus*, *gastrocnemius*, *quadriceps*, *soleus* and *tibialis anterior* muscles, as well as spleen, were removed and weighed. Tibia length was also determined. *Tibialis anterior* muscle was used for histochemical, molecular biology and biochemical analyses. Mice were then killed by cervical dislocation while deeply anaesthetised.

### 2.7 Serum Assay in sActRIIB‐Fc Treated Mice

Sera prepared from blood samples were used for the measurement of circulating myostatin concentration following the manufacturer's instructions (GDF‐8/Myostatin ELISA kit DGDF80, R&D Systems).

### 2.8 Morphometric Analysis of Neuromuscular Junction

*Extensor digitorum longus* muscles from 2‐month‐old WT and KI mice were fixed in 4% paraformaldehyde for 30 min. Muscles were then teased to isolate individual fibres and small fibre bundles. Fibres were incubated overnight at 4°C in PBS with 0.1 M glycine, washed, permeabilized and saturated for 3 h in PBS with 0.2% saponin and 5% goat serum. After washing, primary antibodies (S100, Agilent; Tubb3, BioLegend; SV2, Developmental Studies Hybridoma Bank) were incubated overnight at 4°C. Fibres were washed and incubated with Alexa Fluor 546 and Alexa Fluor 647 secondary antibodies (ThermoFisher Scientific). Nuclei and nicotinic acetylcholine receptors were stained with DAPI and Alexa Fluor 488‐conjugated‐α‐bungarotoxin (ThermoFisher Scientific) for 1.5 h at room temperature. After washing, fibres were mounted on a glass slide with Fluoromount (Invitrogen). Immunofluorescence was analysed using a confocal microscope (Leica SP5). Images were processed using ImageJ software, and morphometric analysis was performed using the ‘NMJ‐morph’ macro [23].

### 2.9 Immunofluorescence and Histochemical Analyses

*Tibialis anterior* muscle sections (10 μm) were prepared using a CM1950 cryostat microtome (Leica). For histomorphometric analysis and detection of nicotinamide adenine dinucleotide tetrazolium reductase (NADH‐TR) abnormalities, sections were saturated with 5% BSA and 10% goat serum for 1 h at room temperature. Sections were then immunostained with a rabbit anti‐laminin α1 primary antibody (1:400, Sigma‐Aldrich), followed by an Alexa Fluor 647 goat anti‐rabbit secondary antibody (1:1000, ThermoFisher Scientific). Sections were then stained with NADH‐TR for 1 h at 37°C. Slides were mounted with Fluoromount (Invitrogen). Images were captured with an Axio Imager 2 microscope equipped with Axiocam 503 and 105 cameras (Zeiss). Muscle cross‐sectional area or minimum Feret diameter was quantified using Fiji (ImageJ). To identify paired box 7 (Pax7)‐positive cells, sections were fixed in 4% paraformaldehyde for 20 min at room temperature, and permeabilized in 100% methanol at −20°C for 6 min. After antigen retrieval (2 × 5 min in 10 mM citrate buffer at 90°C), sections were saturated in 4% BSA for 2 h at room temperature and then co‐labelled overnight at 4°C with anti‐Pax7 (1:50, Developmental Studies Hybridoma Bank) and anti‐laminin α1 (1:100, Sigma‐Aldrich) primary antibodies using PBS with 2% BSA. For the detection of Pax7, sections were incubated for 1 h at 37°C with a biotin‐conjugated secondary antibody (1:200, VectorLabs) followed by the incubation with fluorescein‐streptavidin for 1 h at 37°C (1:1000, Beckman Coulter). A Cy3‐conjugated secondary antibody (1:200, Jackson ImmunoResearch) incubated for 45–60 min at 37°C was used for the detection of laminin. Images were acquired with an Axio Observer Z1 connected to a CoolSNAP HQ2 camera (Zeiss). For pericentriolar material 1 (PCM1) immunostaining of myonuclei, muscle sections were permeabilized with 0.5% Triton X‐100 in PBS for 10 min at room temperature, saturated with 2% BSA in PBS for 1 h at room temperature and then incubated overnight at room temperature with anti‐laminin α2 rat primary antibody (1:1000, Santa Cruz Biotechnology) and anti‐PCM1 rabbit primary antibody (1:1000, Sigma‐Aldrich). Anti‐rat Alexa Fluor 647 (1:1000, ThermoFisher Scientific) and anti‐goat Alexa Fluor 488 (1:1000, ThermoFisher Scientific) secondary antibodies were then incubated in PBS for 1 h at room temperature. Sections were counterstained with Hoechst 3342 (0.2% in PBS) for 2 min at room temperature and mounted with Fluoromount (Invitrogen). Images were acquired with an Axio Imager 2 microscope connected to an Axiocam 503 camera (Zeiss). PCM1‐positive myonuclei per fibre were quantified by manually counting PCM1‐immunoreactive nuclei colocalized with Hoechst nuclear staining.

### 2.10 RNA Extraction, Reverse Transcription and Real‐Time Quantitative Polymerase Chain Reaction Analysis

Total RNA was extracted from powdered *tibialis anterior* muscle by using RNeasy Fibrous Tissue Mini Kit (Qiagen). After mRNA to cDNA conversion using iScript cDNA synthesis kit (Bio‐Rad), PCR was performed in a 20 μL final volume using Takyon No Rox SYBR kit (Eurogentec) using a CFX96 Real‐Time PCR Detection System (Bio‐Rad). Data were analysed using the ∆∆C*t* method of analysis. Reference genes (*Actb, Hprt1* and *Tuba1*) were used for normalization. Selected forward and reverse primer sequences are listed in Table S1.

### 2.11 Protein Extraction and Immunoblotting

Powdered *tibialis anterior* muscles were homogenized (1:20 wt:vol) in a RIPA buffer (Cell Signalling Technology) supplemented with protease and phosphatase inhibitors (Roche). Samples were centrifuged (10 min × 12 000 *g* at 4°C), and the protein concentration of the supernatants was determined (DC protein assay, Bio‐Rad). Thirty micrograms of proteins in Laemmli buffer (Bio‐Rad) were subjected to 12% stain‐free gel electrophoresis (Bio‐Rad) and transferred onto nitrocellulose membrane (Trans‐Blot Turbo Transfer System, Bio‐Rad). Membranes were blocked with a TBS‐0.1% Tween 20 solution containing 5% non‐fat dried milk for 2 h at room temperature and then incubated overnight at 4°C with a cocktail of antibodies against oxidative phosphorylation mitochondrial complexes (1:1000, Abcam), Akt (1:1000, Cell Signalling Technology), Akt^Ser473^ (1:1000, Cell Signalling Technology), P62 (1:1000, Cell Signalling Technology), LC3B (1:1000, Cell Signalling Technology) and FOXO3 (1:1000, Cell Signalling Technology), followed by probing with a StarBright‐conjugated secondary antibody (1:2000, Bio‐Rad). After imaging the membranes (ChemiDoc MP Imaging System, Bio‐Rad), protein band intensity was determined using Image Lab 6.0. Stain‐free blots (Bio‐Rad) were used to check for equal protein loading.

### 2.12 Statistical Analysis

All statistical analyses were performed using GraphPad PRISM 10.4.1. Data are presented as means ± SD Grubb's test was performed to identify outliers. Normality was assessed using the Shapiro–Wilk test. Depending on the data distribution, statistical comparisons were performed using the Mann–Whitney test or an unpaired two‐tailed *t*‐test. For multiple group comparisons, one‐way ANOVA followed by Tukey's multiple comparison test, two‐way ANOVA followed by Tukey's multiple comparison test or two‐way ANOVA with repeated measures followed by Sidak's multiple comparison test were used. Principal component analysis was also performed. Details of the statistical tests applied to each variable are provided in the figure legends. A significance threshold of 0.05 was used for all comparisons.

## 3 Results

### 3.1 Postnatal Skeletal Muscle Growth Is Impaired in KI Mice

**FIGURE 1:**
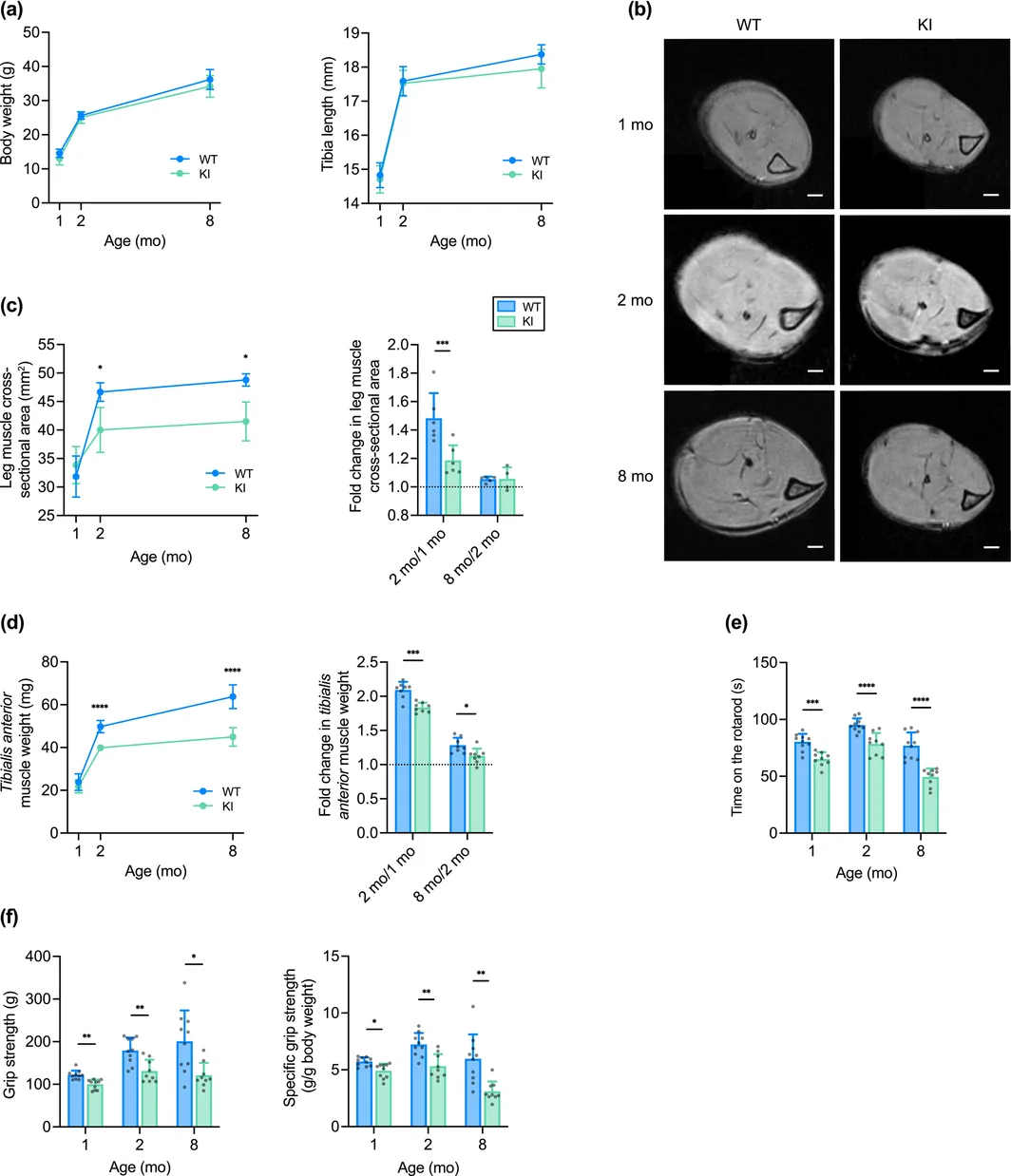
Postnatal skeletal muscle growth is impaired in KI mice. (a) Body weights (left) were similar in WT (*n* = 7–9) and KI (*n* = 7–11) mice at 1, 2 and 8 months (mo) of age. Tibia length (right) indicates similar growth rates between WT (*n* = 7–8) and KI (*n* = 8) mice. (b) Representative magnetic resonance images of leg muscle cross‐sectional area in 1‐, 2‐ and 8‐month‐old WT and KI mice. Scale bar equals 1 mm. (c) Quantification of magnetic resonance images (left) showed impaired skeletal muscle growth between 1 and 2 months of age in KI mice (*n* = 4–6) compared to WT mice (*n* = 5–6). Fold change in leg muscle cross‐sectional area (right) between 1 and 2 months of age (2 mo/1 mo) and 2 and 8 months of age (8 mo/2 mo). (d) *Tibialis anterior* muscle weight (left) in WT (*n* = 8) and KI (*n* = 8) mice at 1, 2 and 8 months of age. Fold change in *tibialis anterior* muscle weight (right) between 1 and 2 months of age (2 mo/1 mo) and 2 and 8 months of age (8 mo/2 mo). (e) The time spent on the rotarod was lower in KI mice (*n* = 9) compared to WT mice (*n* = 10). (f) Absolute (left) and normalized (right) muscle grip strength in KI mice (*n* = 9–10) and WT mice (*n* = 9–10). Data are means ± SD. (a left, c, e and f) Data were analysed by a two‐way ANOVA with repeated measures followed by Sidak's multiple comparison test. (a right and d) Data were analysed by a two‐way ANOVA followed by Tukey's multiple comparison test. *p < 0.05, **p < 0.01, ***p < 0.001 and ****p < 0.0001.

KI mice were viable, fertile, gained weight and grew normally (Figure 1a), as indicated by tibia length. However, MRI analysis revealed significantly lowered muscle mass at 2 and 8 months of age in KI mice, as shown by reduced leg muscle cross‐sectional area (Figure 1b,c). This was due to impaired growth as the KI mice exhibited only a 1.2‐fold increase in muscle cross‐sectional area compared to the 1.5‐fold increase observed in WT mice over the same period (*p* < 0.001). By 8 months, the fold change was close to one in both genotypes. Accordingly, measurement of skeletal muscle weight confirmed reduced muscle growth in KI mice between 1 and 2 months in the *tibialis anterior* (Figure 1d), *quadriceps*, *gastrocnemius* and *extensor digitorum longus* muscles (Figure S1a–c), a defect that was not later compensated. *Soleus* muscle weight was non‐significantly decreased (Figure S1d). Functionally, KI mice displayed impaired motor coordination (Figure 1e) and reduced grip strength (Figure 1f) starting at 1 month, thus preceeding skeletal muscle growth impairment. Morphometric analysis of neuromuscular junctions in the *extensor digitorum longus* muscle at 2 months revealed a slight increase in the amount of postsynaptic acetylcholine receptor. However, no significant pre‐ or postsynaptic morphological abnormalities were observed that could account for the functional deficit (Figure S2).

### 3.2 Histological Abnormalities Are Associated With Worsening Muscle Growth Defect

**FIGURE 2:**
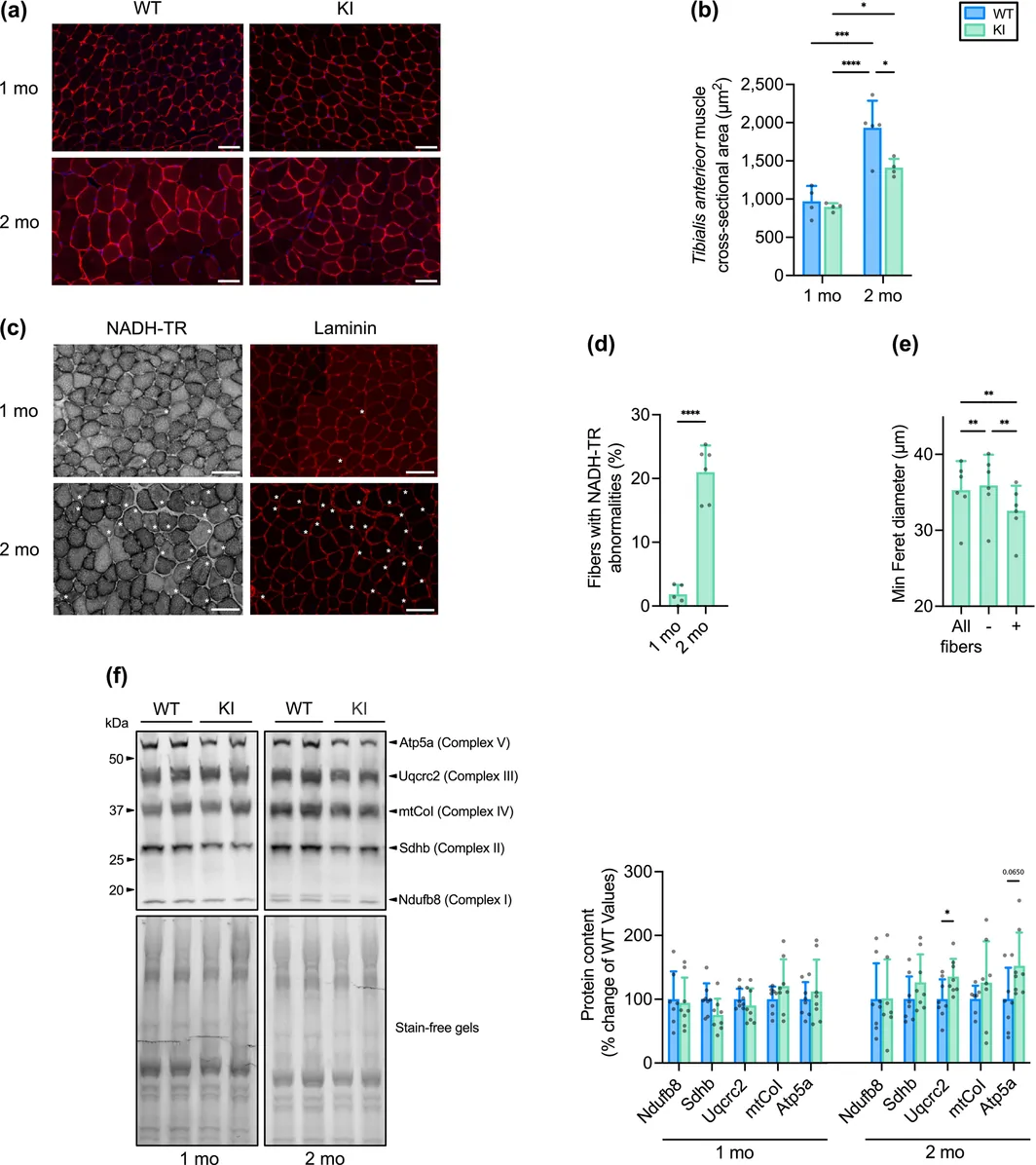
Histological abnormalities are associated with worsening muscle growth defect. (a) Representative images of a cross‐section of *tibialis anterior* muscle immuno‐stained with laminin (red) in WT and KI mice at the age of 1 and 2 months (mo). Scale bar equals 50 μm. (b) Quantification of muscle fibre cross‐sectional area in WT (*n* = 4–5) and KI (*n* = 4) mice. A mean of 537 ± 60 fibres per muscle was analysed. (c) Representative nicotinamide adenine dinucleotide tetrazolium reductase (NADH‐TR) staining and laminin immunostaining (red) of *tibialis anterior* muscle sections of 1‐ and 2‐month‐old KI mice. Scale bar equals 50 μm. (d) The proportion of fibres displaying NADH‐TR staining abnormalities increases between 1 and 2 months of age in KI mice (*n* = 5–6). No NADH‐TR staining abnormality was observed in WT mice. An average of 550 ± 129 positive fibres and 2534 ± 280 negative fibres were counted per muscle. (e) The presence of NADH‐TR staining abnormalities is associated with a lower min Feret diameter in 2‐month‐old KI mice (*n* = 6). A total of 18 507 fibres were analysed including 3302 positive fibres and 15 205 negative fibres. (f) Representative western blot images of oxidative phosphorylation protein expression (left, upper panel). Stain‐free blots (left, lower panel) were used for protein normalization. Protein levels (right) of Ndufb8 (complex I), Sdhb (complex II), Uqcrc2 (complex III), mtCoI (complex IV) and Atp5a (complex V) in WT and KI mice (*n* = 7–8). Data are means ± SD. (b) Data were analysed by a two‐way ANOVA followed by Tukey's multiple comparison test. (d and f) Data were analysed by unpaired *t*‐test or Mann–Whitney test (Atp5a 2 mo). (e) Data were analysed by a one‐way ANOVA followed by Tukey's multiple comparison test. *p < 0.05, **p < 0.01, ***p < 0.001 and ****p < 0.0001.

As the defect in muscle growth occurred between 1 and 2 months of age, we next focused our analyses at these time points. At 2 months, KI mice displayed a 25% reduction in muscle fibre cross‐sectional area compared to WT (Figure 2a,b). The prevalence of the histological hallmark of the disease, i.e., a central NADH‐TR staining pattern, which colocalizes with succinate dehydrogenase and cytochrome oxidase stainings [17], was markedly increased in 2‐month‐old KI mice, affecting more than 20% of KI muscle fibres (Figure 2c,d), suggesting a spatial reorganization of oxidative mitochondrial activity within the myofibres. Furthermore, NADH‐TR‐positive fibres also had smaller minimum Feret diameter compared to NADH‐TR‐negative fibres of KI mice (Figure 2e). Finally, assessment of oxidative phosphorylation protein expression indicated that some mitochondrial proteins showed an upward trend at 2 months, with Uqcrc2 (Complex III) reaching statistical significance (*p* < 0.05) and Atp5a (Complex V) showing a strong trend (*p* = 0.065) (Figure 2f). Collectively, these data suggest that both the altered spatial distribution of oxidative enzymes and the trend to an increased expression of mitochondrial protein complexes reflect the worsening muscle fibre growth defect of KI mice.

### 3.3 Mechanisms Contributing to Skeletal Muscle Growth Impairment

**FIGURE 3:**
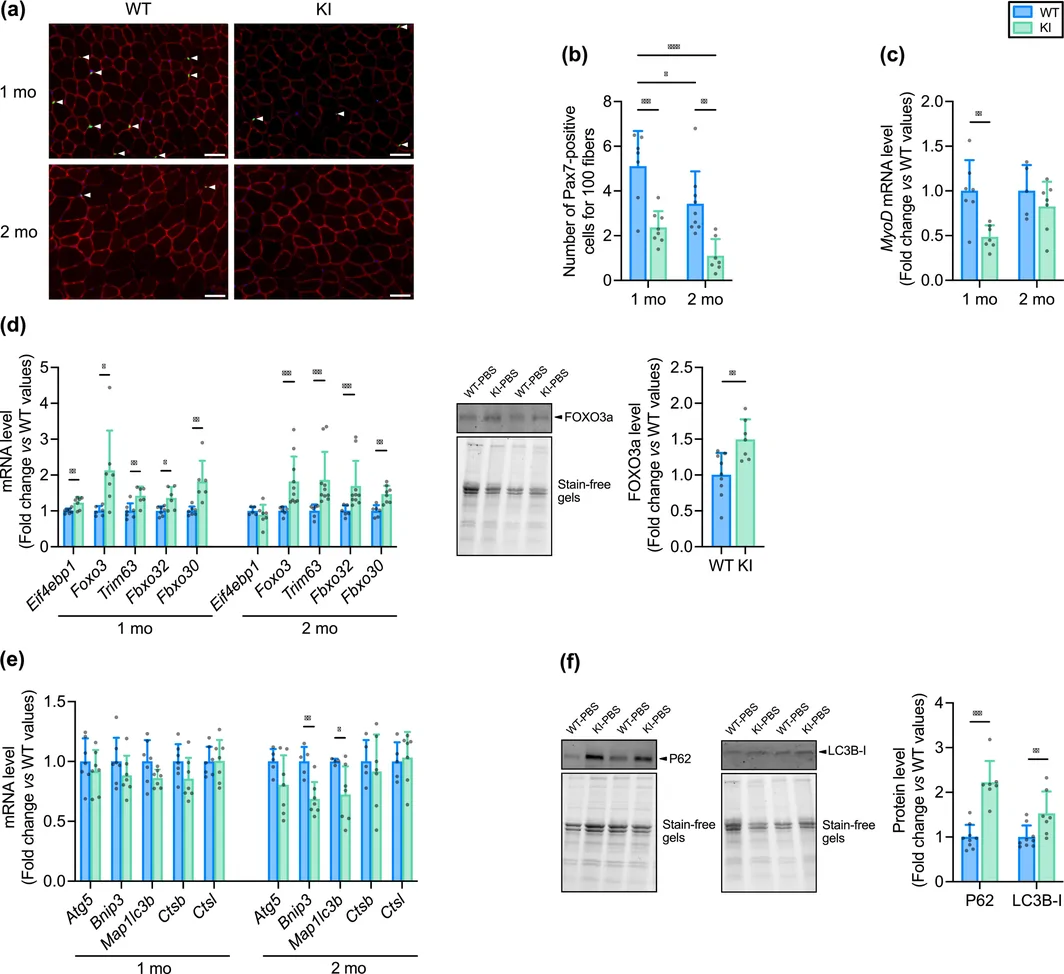
Mechanisms involved in skeletal muscle growth defect of KI mice. (a) Representative laminin (red)/Pax7 (green) immunolabelling of *tibialis anterior* muscle cross‐section in WT and KI mice aged of 1 and 2 months (mo). Scale bars equal 50 μm. (b) The number of Pax7‐positive cells decreases in KI mice (*n* = 7–8) when compared to WT mice (*n* = 7–9). An average of 825 ± 277 fibres was analysed per muscle. (c) MyoD mRNA level in the *tibialis anterior* muscle of WT (*n* = 5–7) and KI (*n* = 7) mice. (d, left) Transcript levels of *Eif4ebp1*, *Foxo3*, *Trim63*, *Fbxo32* and *Fbxo30* in the *tibialis anterior* muscle of WT (*n* = 5–7) and KI (*n* = 6–10) mice. (d, right) Representative immunoblot of FOXO3 and stain‐free gel used for protein normalization. Quantification of FOXO3 protein content in 2‐month‐old WT (*n* = 9) and KI (*n* = 7) mice. (e) Transcripts levels of autophagy‐lysosome pathway related genes in the *tibialis anterior* muscle of WT (*n* = 4–7) and KI (*n* = 7) mice. (f) Representative immunoblots P62 and LC3B‐I and stain‐free gels used for protein normalization. Quantification of P62 and LC3B‐I protein content in 2‐month‐old WT (*n* = 9) and KI (*n* = 6–7) mice. Data are means ± SD (b) Data were analysed by a two‐way ANOVA followed by Tukey's multiple comparison test. (c, d, e and f) Data were analysed by unpaired *t*‐test or Mann–Whitney test (*Foxo3* 2 mo, *Trim63* 2 mo, *Fbxo32* 2 mo). *p < 0.05, **p < 0.01, ***p < 0.001 and ****p < 0.0001.

Postnatal myofibre growth requires the incorporation of new myonuclei derived from Pax7‐positive myogenic precursor cells to sustain the increased transcriptional and translational demand of growing myofibres [24]. In KI mice, the number of Pax7‐positive cells was significantly reduced in the *tibialis anterior* muscle at both 1 and 2 months of age (Figure 3a,b). This was accompanied by decreased *MyoD* mRNA level at 1 month (Figure 3c), together suggesting reduced myonuclear accretion during this period. In parallel, our transcriptional analysis revealed an upregulation of the translational repressor *Eif4ebp1* in 1‐month‐old KI mice (Figure 3d), along with increased mRNA levels of *Foxo3* and E3 ligases *Trim63* (MuRF1), *Fbxo32* (Atrogin‐1) and *Fbxo30* (Musa1) at 1 and 2 months (Figure 3d). FOXO3 protein level was also increased in 2‐month‐old KI mice (Figure 3d). In addition, mRNA levels of key autophagy‐related genes, *Bnip3* and *Map 1lc3b*, were decreased at 2 months (Figure 3e), together with increased protein content of P62 and LC3B‐I at 2 months (Figure 3f), suggesting impaired autophagy flux. Collectively, these data point to disrupted protein homeostasis in 2‐month‐old KI mice.

### 3.4 Myostatin Gene Invalidation in KI Mice Increases Muscle Mass and Restores Muscle Function in KI Mice

**FIGURE 4:**
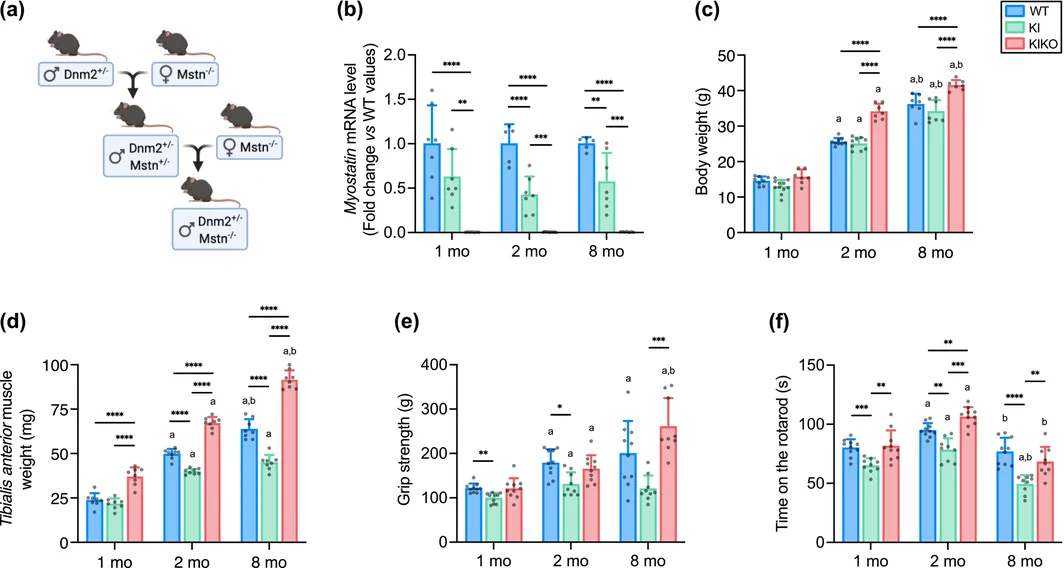
*Myostatin* gene invalidation in KI mice increases skeletal muscle mass and restores muscle function. (a) Schematic view of the breeding strategy to generate KI mice lacking exon 3 of the *myostatin* (*Mstn*) gene (KIKO mice). (b) *Myostatin* mRNA level in WT (*n* = 5–7), KI (*n* = 7) and KIKO (*n* = 7–9) mice at the age of 1, 2 and 8 months (mo). Transcript was undetectable in KIKO mice. (c) Body weight of WT (*n* = 7–9), KI (*n* = 7–11) and KIKO (*n* = 7) mice. (d) *Myostatin* gene invalidation increases *tibialis anterior* muscle weight (*n* = 8). (e) *Myostatin* gene invalidation restores muscle grip strength (WT, KI, KIKO: *n* = 9–10). (f) *Myostatin* gene invalidation restores rotarod performance (WT, KI, KIKO: *n* = 9–10). Data of WT and KI mice have been previously presented in Figure 1. Data are means ± SD (b) Data were analysed with a one‐way ANOVA followed by Tukey's multiple comparison test. (c, e and f) Data were analysed by a two‐way ANOVA with repeated measures followed by Sidak's multiple comparison test. (d) Data were analysed by a two‐way ANOVA followed by Tukey's multiple comparison test. *p < 0.05, **p < 0.01, ***p < 0.001 and ****p < 0.0001. a, significantly different from corresponding 1‐month‐old mice. b, significantly different from corresponding 2‐month‐old mice.

Given the effects of myostatin on muscle anabolism inhibition [10] and myoblast proliferation and differentiation [12, 25, 26], we investigated whether its inhibition could improve the phenotype of KI mice. KI mice lacking exon 3 of the *myostatin* gene (KIKO mice) were thus generated (Figure 4a). *Myostatin* transcript level was undetectable in skeletal muscle of KIKO mice and reduced in KI mice compared to WT (Figure 4b). KIKO mice exhibited normal growth (Figure S3a) and significantly higher body weight at 2 and 8 months of age (Figure 4c) compared to age‐matched WT and KI mice. This was accompanied by a substantial increase in *tibialis anterior* (Figure 4d), *quadriceps*, *extensor digitorum longus* and *soleus* muscle mass (Figure S3b–d) that far exceeded that of WT mice. Functionally, KIKO mice showed full restoration of grip strength (Figure 4e) and motor coordination (Figure 4f). Together, these data provide the proof of concept that *myostatin* gene invalidation is an efficient strategy to improve muscle function of KI mice.

### 3.5 Pharmacological Inhibition of Myostatin/Activin A Restores Muscle Mass in KI Mice

**FIGURE 5:**
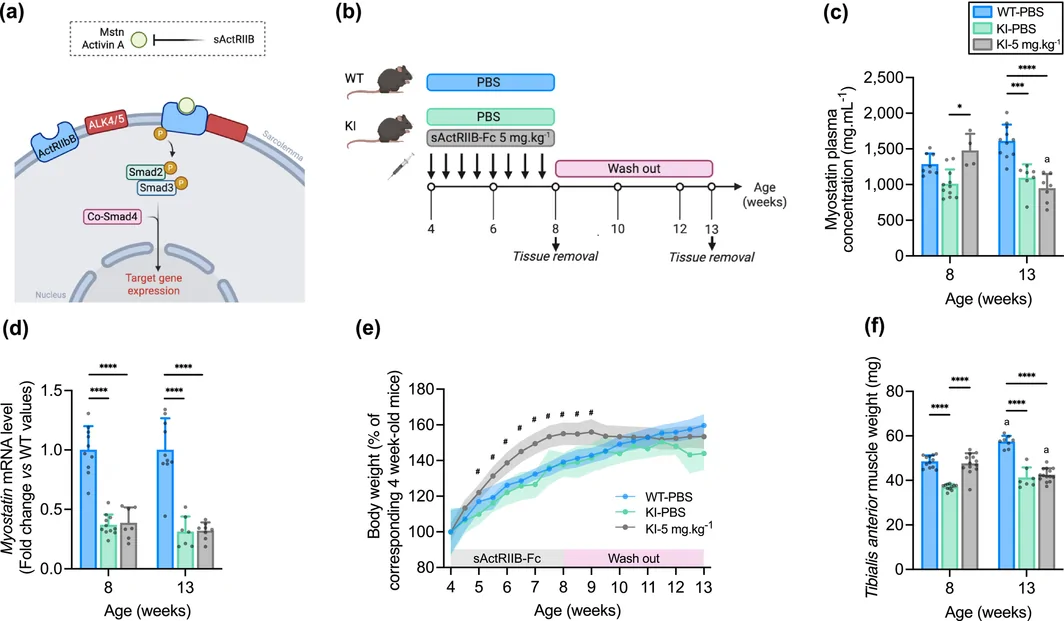
Pharmacological inhibition of myostatin/activin A restores muscle mass in KI mice. (a) Schematic view of the myostatin (Mstn) signalling pathway highlighting the inhibitory effect of soluble activin type IIB receptor (sActRIIB‐Fc). (b) Schematic view of the experimental design. KI mice were treated twice a week by intraperitoneal injections of sActRIIB‐Fc at the dosage of 5 mg kg^−1^ starting at 4 weeks of age for four consecutive weeks. Mice were then maintained in the animal facility five more weeks without any treatment (washout). Tissues were removed either at the end of the treatment (8 weeks of age) or 5 weeks later (13 weeks of age). (c) Myostatin plasma concentration in WT‐PBS (*n* = 8–11), KI‐PBS (*n* = 7–11) and KI‐5 mg kg^−1^ (*n* = 4–7) mice. (d) *Myostatin* mRNA level in the *tibialis anterior* muscle of WT‐PBS (*n* = 10), KI‐PBS (*n* = 7–11) and KI‐5 mg kg^−1^ (*n* = 7–8) mice. (e) Body weight of WT‐PBS (*n* = 8–13), KI‐PBS (*n* = 8–11) and KI‐5 mg kg^−1^ (*n* = 9–14) mice. (f) *Tibialis anterior* muscle weight of WT‐PBS (*n* = 8–12), KI‐PBS (*n* = 7–11) and KI‐5 mg kg^−1^ mice (*n* = 13–14) mice. Data are means ± SD (c and f) Data were analysed with a two‐way ANOVA followed by Tukey's multiple comparison test. (d) Data were analysed with a one‐way ANOVA followed by Tukey's multiple comparison test. (e) Data were analysed by a two‐way ANOVA with repeated measures followed by Sidak's multiple comparison test. *p < 0.05, ***p < 0.001 and ****p < 0.0001. #, significantly different from age‐matched WT‐PBS and KI‐PBS mice. a, significantly different from corresponding 8‐week‐old mice.

We next sought to determine whether a pharmacological intervention could reverse an established phenotype. We used a soluble activin type IIB receptor linked to IgG1 Fc fragment (sActRIIB‐Fc), a decoy receptor that sequesters myostatin and activin A (Figure 5a) [27]. KI mice first received intraperitoneal injections of sActRIIB‐Fc at 1, 5 and 10 mg.kg^−1^ twice weekly for 4 weeks starting at 8 weeks of age (Figure S4a). The treatment rapidly increased body weight over the values of WT‐PBS mice, with the 5 and 10 mg.kg^−1^ groups showing the greatest gains (Figure S4b). At 5 and 10 mg.kg^−1^, *tibialis anterior* muscle weight was partially restored, *quadriceps* mass was completely restored, and *gastrocnemius* muscle mass exceeded WT values (Figure S4c). We selected the 5 mg.kg^−1^ dosage for further experiments.

Four‐week‐old KI mice were treated with 5 mg kg^−1^ sActRIIB‐Fc (KI‐5 mg kg^−1^) twice weekly for 4 weeks (Figure 5b) and analysed either at the end of the treatment (8 weeks of age) or after a 5‐week washout period (13 weeks of age). Circulating myostatin level was elevated in KI‐5 mg kg^−1^ mice at 8 weeks but returned to KI‐PBS values by the end of the washout period (Figure 5c). Conversely, *myostatin* mRNA level remained low in both KI‐PBS and KI‐5 mg kg^−1^ mice (Figure 5d). Spleen weight, an indicator of immune response, increased during treatment but normalized after the washout period (Figure S5a). Treated mice showed normal growth (Figure S5b) and body weight gain (Figure 5e). Body weight plateaued upon treatment cessation (8 weeks) and returned to WT‐PBS and KI‐PBS values within 2 weeks after the treatment ended. sActRIIB‐Fc treatment induced a marked anabolic effect, significantly increasing muscle mass (Figures 5f and S5c,d). However, this effect proved to be entirely reversible: by the end of the 5‐week washout period, muscle mass had returned to the baseline levels observed in KI‐PBS mice (Figures 5f and S5c,d). Together, these data indicate that while sActRIIB‐Fc effectively restores muscle mass, continuous treatment is required to maintain a sustainable effect.

### 3.6 Mechanisms Underlying the Hypertrophic Effect of sActRIIB‐Fc

**FIGURE 6:**
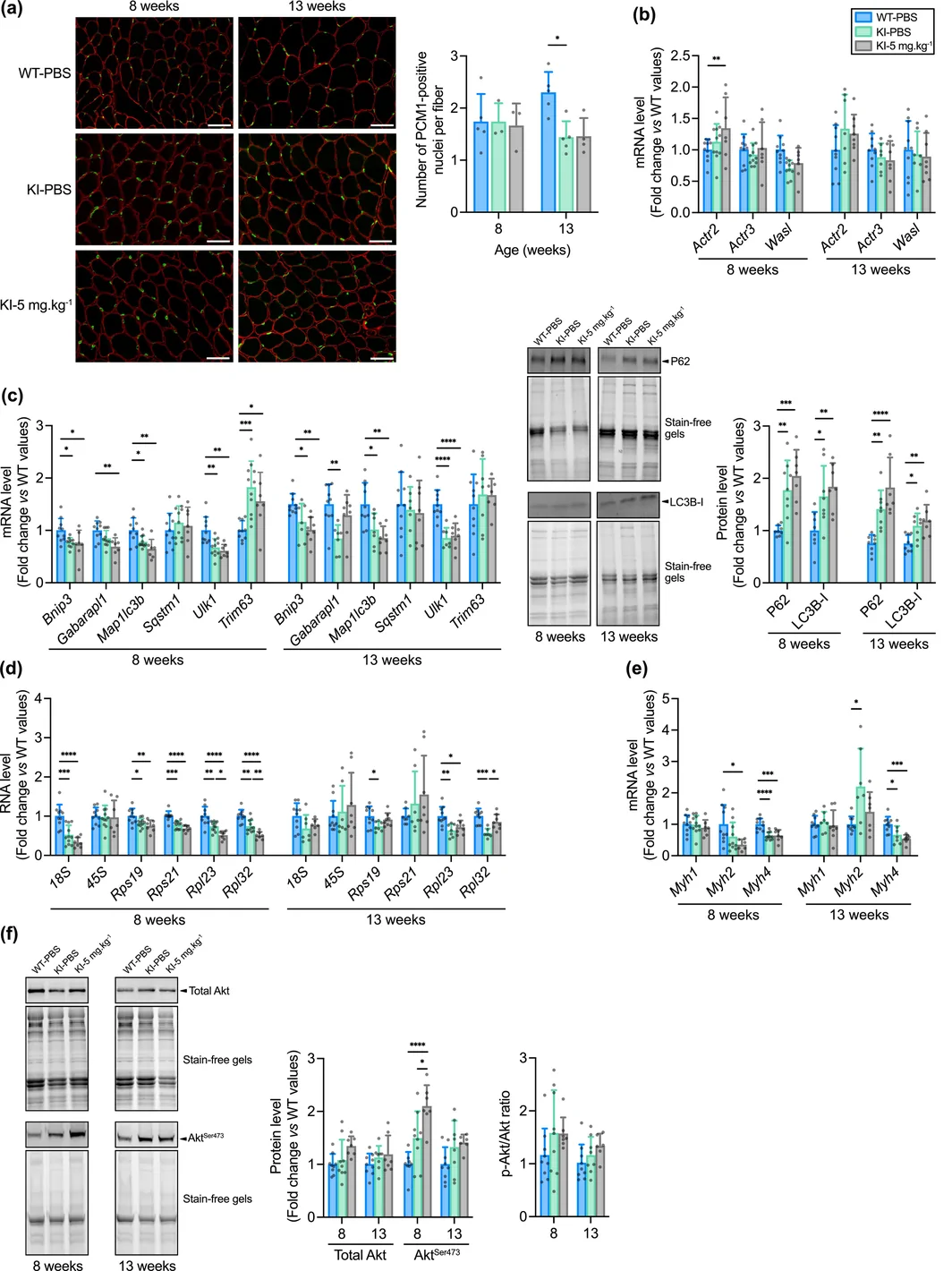
Mechanisms underlying the hypertrophic effect of soluble activin type IIB receptor. (a) Immunostaining (left) representative of pericentriolar material 1 (PCM1) immunostaining (green) counter‐stained with laminin (red) in WT‐PBS, KI‐PBS and KI‐5 mg kg^−1^ mice at 8 and 13 weeks and number of PCM1‐positive nuclei per fibre in the *tibialis anterior* muscle (right) of WT‐PBS mice (*n* = 5), KI‐PBS mice (*n* = 3–5) and KI‐5 mg kg^−1^ mice (*n* = 3–4). A mean of 736 ± 321 fibres per muscle was counted. (b) *Actr2*, *Actr3* and *Wasl* mRNA level in the *tibialis anterior* muscle of WT‐PBS (*n* = 10), KI‐PBS (*n* = 7–11) and KI‐5 mg.kg^−1^ (*n* = 6–8) mice. (c, left) Transcripts levels of components of autophagy‐lysosome pathway in the *tibialis anterior* muscle (left) of WT‐PBS (*n* = 9–10), KI‐PBS (*n* = 7–11) and KI‐5 mg.kg^−1^ (*n* = 7–8) mice and for *Trim63* mRNA level in *tibialis anterior* muscle (right) of WT‐PBS mice (*n* = 10), KI‐PBS mice (*n* = 7–11) and KI‐5 mg kg^−1^ mice (*n* = 7). (c, right) Representative immunoblots of P62 and LC3B‐I and stain‐free gels used for protein normalization. Quantification of P62 and LC3B‐I protein content in 2‐month‐old WT (*n* = 8–9), KI‐PBS (*n* = 8–9), and KI‐5 mg.kg^−1^ (*n* = 7) mice. (d) Transcript levels of *Myh1*, *Myh2*, and *Myh4* in the *tibialis anterior* muscle of WT‐PBS (*n* = 9–10), KI‐PBS (*n* = 7–11) and KI‐5 mg kg^−1^ (*n* = 7–8) mice. (e) Transcript levels of rRNA (*18S* and *45S*) and mRNA of ribosomal subunits (*Rps19* and *Rps21*, *Rpl23* and *Rpl32*) in the *tibialis anterior* muscle of WT‐PBS (*n* = 8–10), KI‐PBS (*n* = 7–11) and KI‐5 mg.kg^−1^ (*n* = 6–8) mice. (f) Representative immunoblots of Akt and Akt^Ser473^ and corresponding stained‐free gels. Quantitative analysis of immunoblot signals of Akt and Akt^Ser473^ in WT‐PBS (*n* = 8–9), KI‐PBS (*n* = 8–9) and KI‐5 mg kg^−1^ (*n* = 6–7) mice. Data are means ± SD (a) Data were analysed with a two‐way ANOVA followed by Tukey's multiple comparison test. (b, c, d, e and f) Data were analysed with a one‐way ANOVA followed by Tukey's multiple comparison test. *p < 0.05, **p < 0.01, ***p < 0.001 and ****p < 0.0001.

Based on the known biological functions of myostatin, we first hypothesized that sActRIIB‐Fc promotes nuclear accretion to drive muscle fibre growth. We used pericentriolar material‐1 (PCM1) immunostaining as a specific marker for myonuclei [20]. While the number of PCM1 positive nuclei per fibre was comparable between groups at 8 weeks of age, this number had decreased in both KI‐PBS and KI‐5 mg kg^−1^ mice at 13 weeks (Figure 6a). This suggests a defect in nuclear accretion in KI mice that is not rescued by sActRIIB‐Fc treatment. Expression of actin cytoskeleton regulators, *Actr2* (Arp2), *Actr3* (Arp3) and *Wasl* (Wasp), involved in myoblast fusion, remained unchanged in both groups of KI mice (Figure 6b). Together, these results suggest that muscle hypertrophy induced by sActRIIB‐Fc at 8 weeks does not result from the additional fusion of muscle precursor cells.

Transcript levels of the E3 ligase *Trim63*, elevated in KI‐PBS mice, remained elevated in KI‐5 mg kg^−1^mice at 8 weeks (Figure 6c left). Transcript levels of autophagy‐related genes (*Bnip3*, *Gabarapl1*, *Map1lc3b, Sqstm1*, *Ulk1*), downregulated in KI‐PBS mice, remained lowered by the treatment (Figure 6c left), whereas the protein contents of P62 and LC3B‐I, which were increased in KI‐PBS mice, remained elevated in KI‐5 mg kg^−1^ at 8 and 13 weeks (Figure 6c right), suggesting persistence of impaired autophagy flux in KI‐5 mg kg^−1^ mice. Similarly, rRNA (*18S*, *45S*) and mRNA of ribosomal proteins (*Rps19*, *Rps21*, *Rpl23* and *Rpl32*) were downregulated in both KI‐PBS and KI‐5 mg kg^−1^ mice at 8 and 13 weeks (Figure 6d). The pool of translatable mRNA encoding myosin heavy chain isoform IIa (*Myh2*) and IIb (*Myh4*), which is necessary to support the synthesis of myofibrillar proteins, was similarly down‐regulated in both groups of KI mice at 8 and 13 weeks (Figure 6e). Finally, immunoblot analyses revealed an increase of Akt^Ser473^ phosphorylation in KI‐5 mg kg^−1^ mice compared to KI‐PBS mice at 8 weeks (Figure 6f), indicative of enhanced Akt signalling. Notably, these effects were reversed after treatment cessation. Together, these data suggest that the anabolic response triggered by sActRIIB‐Fc treatment was primarily associated with an activation of regulatory mechanisms controlling protein synthesis, rather than with the regulation of proteolytic pathways or transcriptional mechanisms governing myofibrillar protein expression.

### 3.7 sActRIIB‐Fc Treatment Does Not Restore Skeletal Muscle Force of KI Mice

**FIGURE 7:**
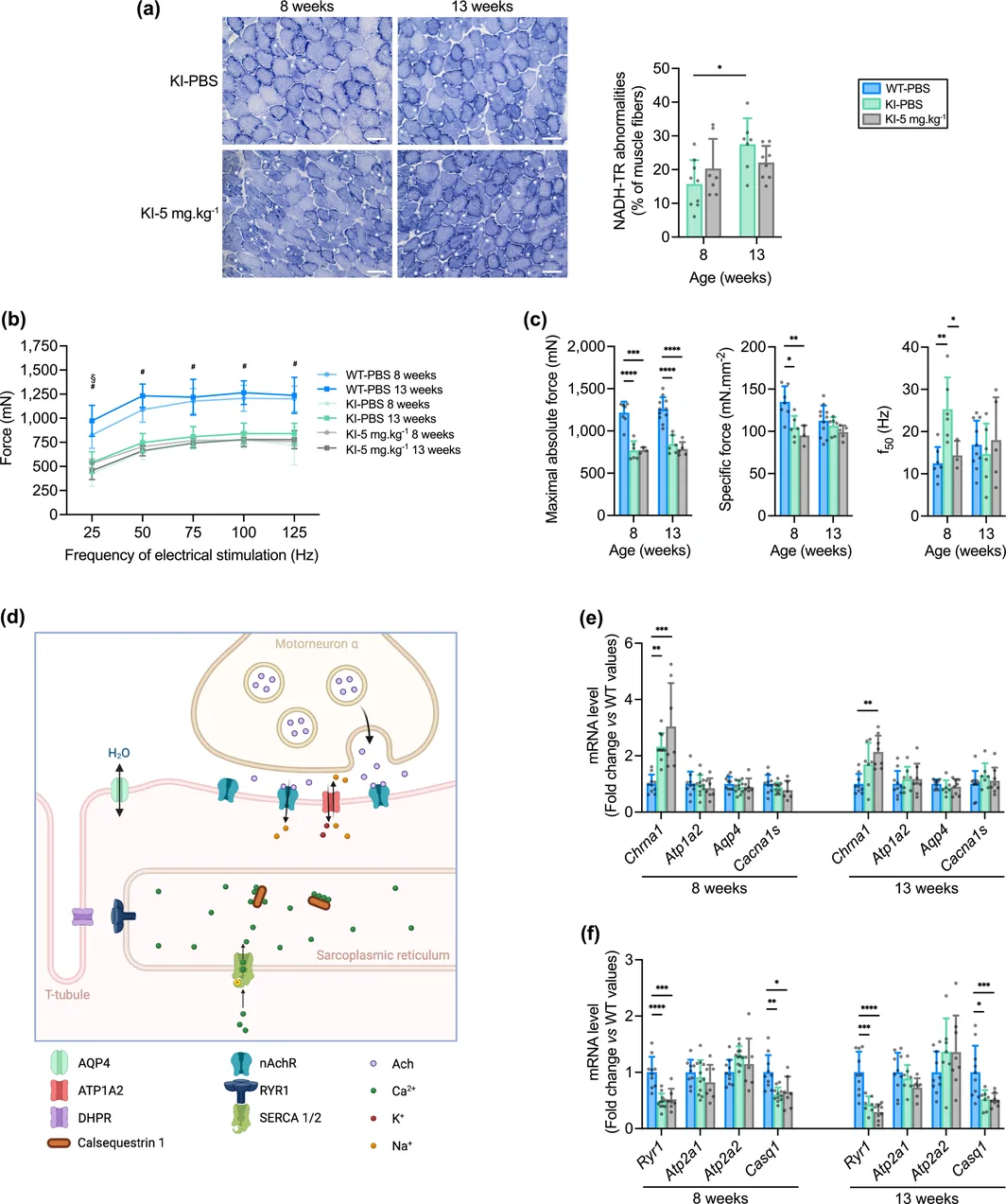
Soluble activin type IIB receptor treatment does not restore skeletal muscle force of KI mice. (a) Representative nicotinamide adenine dinucleotide tetrazolium reductase (NADH‐TR) staining of *tibialis anterior* muscle cross‐sections (left) of KI‐PBS and KI‐5 mg kg^−1^ mice at 8 and 13 weeks were asterisks show fibres with abnormal NADH‐TR staining and the number of fibres with NADH‐TR staining abnormalities (right) in KI‐5 mg kg^−1^ (*n* = 7–8) compared to KI‐PBS (*n* = 7–9); a mean of 2804 ± 317 fibres was analysed per muscle. *Tibialis anterior* muscle fibres from WT mice did not show any NADH‐TR staining abnormalities and were therefore not represented. (b) Muscle force‐frequency relationship in WT‐PBS (*n* = 7–11), KI‐PBS (*n* = 6) and KI‐5 mg kg^−1^ (*n* = 3–5) mice at 8 and 13 weeks. (c) Maximal absolute isometric force (F_max_), specific isometric force (F_s_), and f_50_ (stimulation frequency at which 50% of F_max_ is achieved) of *tibialis anterior* muscle in WT‐PBS (*n* = 7–11), KI‐PBS (*n* = 6) and KI‐5 mg kg^−1^ (*n* = 3–5) mice at 8 and 13 weeks. (d) Schematic view of the molecular targets analysed. *Chrna1* encodes the alpha1 subunit of the nicotinic acetylcholine (Ach) receptor (nAchR). *Aqp4* encodes aquaporin 4 that regulates water movement across the sarcolemma. *Atp1a2* encodes the Na^+^/K^+^‐transporting ATPase subunit alpha‐2. *Cacna1s* encodes the voltage‐dependent L‐type Ca^2+^ channel subunit alpha‐1S of the dihydropyridine receptor (DHPR). *Ryr1* (ryanodine receptor 1) is a Ca^2+^ release channel of the sarcoplasmic reticulum that opens in response to depolarization. *Atp2a1* and *Atp2a2* encode the sarcoplasmic/endoplasmic reticulum Ca^2+^ ATPase fast (SERCA1) and slow (SERCA2) isoforms, respectively. *Casq1* encodes calsequestrin 1, a reticulum sarcoplasmic Ca^2+^ buffer protein. (e) *Chrna1*, *Atp1a2*, *Aqp4* and *Cacna1s* mRNA levels in the *tibialis anterior* muscle of WT‐PBS (*n* = 9–10), KI‐PBS (*n* = 7–11) and KI‐5 mg kg^−1^ (*n* = 6–8) mice. (f) *Ryr1*, *Atp2a1*, *Atp2a2* and *Casq1* mRNA levels in the *tibialis anterior* muscle of WT‐PBS (*n* = 9–10), KI‐PBS (*n* = 7–11), and KI‐5 mg kg^−1^ (*n* = 7–8) mice. Data are means ± SD (a and c) Data were analysed with a two‐way ANOVA followed by Tukey's multiple comparison test. (b) Data were analysed with a two‐way ANOVA with repeated measures followed by Sidak's multiple comparison test. (e and f) Data were analysed with a one‐way ANOVA followed by Tukey's multiple comparison test. *p < 0.05, **p < 0.01, ***p < 0.001 and ****p < 0.0001. #, significantly different from WT‐PBS at 8 and 13 weeks. a: significantly different from corresponding 8‐weeks‐old WT‐PBS mice. §: significantly different from corresponding 50, 75, 100 and 125 Hz.

sActRIIB‐Fc treatment did not lower the proportion of muscle fibres exhibiting NADH‐TR staining abnormalities (Figure 7a). sActRIIB‐Fc treatment also did not restore the force produced in situ by the *tibialis anterior* muscle at 8 weeks regardless of the frequency of stimulation (Figure 7b). Absolute and specific maximal isometric forces (F_max_ and F_s_, respectively) were not improved by the treatment (Figure 7c). Interestingly, KI‐PBS mice required an approximately twofold higher stimulation frequency to reach 50% of their maximal force compared to WT controls (Figure 7c). Conversely, a significant improvement was observed in response to sActRIIB‐Fc treatment, as treated KI mice exhibited significantly restored f_50_ values at 8 weeks (Figure 7c). To gain further insights into the mechanisms involved in the loss of muscle force in KI‐PBS and KI‐5 mg.kg^−1^ mice, we examined the expression of genes regulating excitation‐contraction coupling (Figure 7d). Transcript levels of *Atp1a2*, *Aqp4*, *Cacna1s*, *Atp2a1* and *Atp2a2* were similar between groups (Figure 7e,f). However, the expression of *Chrna1*, *RyR1* and *Casq1* was altered both in KI‐PBS and KI‐5 mg.kg^−1^ mice. These findings suggest that altered expression of key excitation‐contraction coupling components (*Chrna1*, *RyR1* and *Casq1*) may likely contribute to the reduced muscle force in KI mice, and that these defects were not alleviated by sActRIIB‐Fc treatment.

## 4 Discussion

We present a comprehensive molecular and functional analysis of both genetic and pharmacological strategies targeting myostatin to counteract skeletal muscle deconditioning caused by the R465W *DNM2* heterozygous mutation. Our findings provide proof of concept that myostatin inhibition can restore the postnatal skeletal muscle growth defect in KI mice. However, the inability of pharmacological inhibition to improve muscle force highlights a critical limitation of targeting muscle mass alone, underscoring the need for therapeutic strategies that restore both muscle mass and function.

MRI and morphological analyses revealed a marked impairment of postnatal skeletal muscle growth in KI mice between 1 and 2 months of age. This defect was more pronounced in fibres exhibiting abnormal NADH‐TR staining, a histological feature previously described in this mouse model [17] and in ADCNM patients [23, 24, 28]. Mechanistically, this growth defect appears to arise from a concerted disruption of several homeostatic pathways. The reduction in Pax7‐positive cells and *MyoD* expression points to a defect in satellite cell‐mediated myonuclear accretion, a process essential during early postnatal growth to support the transcriptional demands of rapidly growing [29]. This also likely explains the reduction in myonuclear number previously reported in adult KI mice [30]. In parallel, the upregulation of FOXO3 and its downstream targets, the E3 ligases *Trim63* and *Fbxo32*, suggests enhanced myofibrillar protein degradation. Similar observations have been reported in a preclinical model of XLCNM [31], suggesting that activation of the ubiquitin‐proteasome pathway may represent a shared driver of hypotrophy amongCNM. The concomitant increase in the mRNA level of the translational repressor *Eif4ebp1* also suggests protein synthesis repression. Although FOXO3 is also known to promote autophagy‐related gene expression, the observed reduction in key autophagy transcripts indicates a disconnection between upstream signalling and effective late‐stage autophagy, consistent with impaired autophagic flux. This defect may lead to the accumulation of undegraded substrates and contribute to mitochondrial abnormalities. Collectively, these data suggest that the KI phenotype results from a global disruption of muscle homeostasis rather than a single defective pathway.

The reduced *myostatin* mRNA level in skeletal muscle of KI mice is consistent with reports of decreased circulating myostatin in neuromuscular diseases [32]. Rather than reflecting primary dysregulation, this reduction likely represents an adaptation to the hypotrophic state. Because myostatin functions as a chalone, i.e., a secreted factor whose expression scales with muscle mass [11], its downregulation in KI mice may act to limit further muscle loss. In this context, genetic inactivation of *myostatin* fully rescued skeletal muscle growth and strength, in agreement with previous studies showing its therapeutic potential in various neuromuscular conditions (reviewed in Nielsen et al. [14]) and muscle wasting conditions [18, 19]. Although not directly investigated here, enhanced satellite cell activation and enhanced myoblast proliferation, previously reported in response to myostatin gene inactivation [12], likely contribute to the improved muscle phenotype observed in KIKO mice, where myostatin is absent during embryogenesis.

Pharmacological inhibition using sActRIIB‐Fc induced a robust hypertrophic response, leading to complete restoration of muscle mass at 8 weeks, agreeing with previous studies reporting increased muscle mass in several models of neuromuscular diseases following administration of sActRIIB, either as a soluble protein or through adeno‐associated virus‐mediated delivery [14]. This hypertrophy was associated with elevated serum myostatin levels, an observation consistent with the pharmacological action of sActRIIB‐Fc and the biological regulation of myostatin production: binding of circulating myostatin to sActRIIB‐Fc may create a sActRIIB‐myostatin complex that remains in circulation longer than free myostatin, leading to increased total serum concentration; furthermore, because myostatin is a chalone [11], its production may be further increased by the newly expanded muscle mass. At the molecular level, the hypertrophic response at 8 weeks was primarily associated with activation of Akt signalling, suggesting preserved anabolic capacity. However, this response remained remarkably restricted. Core pathological molecular features of the KI phenotype, including upregulation of E3 ligase, impaired autophagy and mitochondrial abnormalities, were not corrected by sActRIIB‐Fc. This dissociation indicates that broader regulation mechanisms governing skeletal muscle homeostasis remain disrupted in KI mice.

In contrast to KIKO mice, the gain in muscle mass proved remarkably unstable with sActRIIB‐FC treatment, with muscle mass returning to baseline KI‐PBS levels within 5 weeks after treatment cessation, indicating that continuous administration is required to sustain muscle mass gain. The difference in outcomes between genetic and pharmacological myostatin inhibition highlights the importance of treatment context. In KIKO mice, myostatin suppression is lifelong and integrated into developmental and postnatal muscle growth programs [8, 12], resulting in stable and functional improvements. In contrast, pharmacological inhibition is transient and limited to the treatment period, producing short‐term hypertrophy without durably modifying the underlying developmental or homeostatic programs necessary for long‐term muscle maintenance. This distinction may explain why continuous administration is required to maintain muscle mass and why functional recovery remains incomplete.

A striking observation was the complete dissociation between the gain in muscle mass and muscle performance with sActRIIB treatment, indicating that increasing muscle size alone is insufficient to restore functional capacity. While sActRIIB‐Fc treatment has been shown to improve muscle strength in other neuromuscular disease models, such as Duchenne muscular dystrophy [33, 34, 35] and spinal muscular atrophy [36], no such improvement was observed in mouse models of XLCNM [15, 16] or nemaline myopathy [37], suggesting that functional recovery with sActRIIB‐Fc treatment may depend on disease‐specific context. In ADCNM [38, 39], but also in XLCNM [31] and ARCNM [40], muscle weakness is also not solely attributable to reduced muscle mass but also involves defects in calcium handling and excitation‐contraction coupling. This is consistent with the established role of Dnm2 in *t*‐tubule organization [24, 41] and with our data of dysregulated expression of excitation‐contraction coupling genes (*Chrna1*, *RyR1*, *Casq1*) both in KI‐PBS and KI‐5 mg kg^−1^ mice. Disruption of these pathways likely limits force generation, even when muscle mass is restored. This inability to restore muscle function indicates that stimulating muscle hypertrophy alone is insufficient if the underlying contractile apparatus and mitochondrial network remain disorganized.

Finally, the dual inhibition of myostatin and activin A achieved with sActRIIB‐Fc may be relevant for human applications, as activin A, which also signals through SMAD2/3 signalling, circulates at higher levels in humans than in mice [42]. Such combined inhibition of myostatin and activin A may offer greater clinical benefits than myostatin inhibition alone. Therefore, myostatin/activin A inhibition emerges as a promising option, which may be combined with other drugs to better address the multifactorial nature of muscle weakness in ADCNM.

## 5 Conclusion

In conclusion, both genetic and pharmacological strategies provide proof of concept that targeting myostatin can restore muscle mass in a preclinical model of ADCNM. However, the inability of pharmacological inhibition to reproduce the sustained and functional improvements of genetic inactivation underscores a major limitation and indicates that this approach does not correct the underlying pathophysiological mechanisms of the disease. Therefore, muscle hypertrophy alone is insufficient to restore muscle function when structural, metabolic and excitation–contraction coupling defects persist. Accordingly, therapeutic strategies should not be evaluated solely based on their anabolic effects, but also on their ability to sustainably restore muscle performance notably beyond the duration of the treatment. This highlights the need for combinatorial approaches that enhance muscle mass while simultaneously improving contractile function.

## Funding

This work was supported by the AFM‐Téléthon through the strategic MyoNeurALP programme.

## Conflicts of Interest

The authors declare no conflicts of interest.

## Supporting information


**Table S1:** List of primers used for qCR analysis. *18S*: 18S ribosomal RNA; *45S*: 45S ribosomal RNA; Eif4ebp1: Eukaryotic translation initiation factor 4E‐binding protein; *Acta1*: Actin alpha; Actb: Actin beta; Actr2: Actin‐related protein 2 (Arp2); *Actr3*: Actin‐related protein 3 (Arp3); Aqp4: Aquaporin 4; *Atg5*: Autophagy related 5; *Atp1a2*: Sodium/potassium‐transporting subunit alpha‐2; *Atp2a1* and *2*: Sarcoplasmic/endoplasmic reticulum calcium ATPase 1 and 2 (SERCA1 and SERCA2); *Bnip3*: BCL2/adenovirus E1B 19 kDa protein‐interacting protein 3; *Cacna1S*: Voltage‐gated L‐type calcium channel subunit alpha‐1S; *CASQ1*: Calsequestrin‐1; *Chrna1*: Acetylcholine receptor subunit alpha‐1; *Ctsb*: Cathepsin B; *Ctsl*: Cathepsin L; *Foxo3*: Forkhead box protein O3; *Fbxo30*: F‐box protein 30 (Musa1); Fbxo32: F‐box only protein 32 (MAFbx); *Gabarapl1*: GABA type A receptor associated protein like 1; *Hprt1*: Hypoxanthine phosphoribosyltransferase 1; Map 1lc3b: Microtubule associated protein 1 light chain 3 beta; *Mstn*: myostatin; *Myh1*, *Myh2* and *Myh4*: Myosin heavy chain 1, 2 and 4 (MHC2X, MHC2A and MHCIIB, respectively); *Myod1*: Myogenic differentiation 1; *Rps19:* Small ribosomal subunit protein S19; *Rps21:* Small ribosomal subunit protein S21; *Rpl23:* Large ribosomal subunit protein S23; *Rpl32:* Large ribosomal subunit protein S32; *Ryr1*: Ryanodine receptor 1; *Sqstm1*: Sequestosome 1; *Trim63*: Tripartite motif containing 63 (MuRF1); *Tuba1*: Tubulin alpha 1; *Ulk1*: Unc‐51 like autophagy activating kinase 1; *Wasl*: WASP like actin nucleation promoting factor.

**Figure S1:** (related to Figure 1). Postnatal skeletal muscle growth is impaired in KI mice. (a) *Quadriceps*, (b) *gastrocnemius*, (c) *extensor digitorum longus* and (d) *soleus* muscle weights in WT (*n* = 6–8) and KI (*n* = 8) mice at 1, 2 and 8 months (mo) of age. Data are means ± SD (a, b, c and d) Data were analysed with a two‐way ANOVA followed by Tukey's multiple comparison test. *p < 0.05, **p < 0.01 and ****p < 0.0001.

**Figure S2:** (related to Figure 1). Morphometric analysis of neuromuscular junction. (a) Representative images of S100 protein (purple), tubulin b3/SV2 (red), a‐bungarotoxin (BTX, green) and merged (nuclei in blue) immunolabelling of *extensor digitorum longus* muscle fibres of 2‐month‐old WT and KI mice. Scale bar equals 50 μm. (b) Presynaptic morphometric parameters. (c) Postsynaptic morphometric parameters. Data are means ± SD in WT (*n* = 5) and KI (*n* = 5) mice at 2 months of age. All data were analysed by unpaired *t*‐test.

**Figure S3:** (related to Figure 4). *Myostatin* gene invalidation increases skeletal muscle mass. (a) *Tibia* length in WT (*n* = 7–9), KI (*n* = 8–11) and KIKO (*n* = 7–9) mice at 1, 2 and 8 months of age. (b) *Quadriceps*, (c) *extensor digitorum longus* and (d) *soleus* muscle weights in WT (*n* = 7–9), KI (*n* = 8–11) and KIKO (*n* = 7–9) mice at 1, 2 and 8 months of age. Data are means ± SD (a, b, c and d) Data were analysed by a two‐way ANOVA followed by Tukey's multiple comparison test. *p < 0.05, **p < 0.01, ***p < 0.001 and ****p < 0.0001. a, significantly different from corresponding 1‐month‐old mice. b, significantly different from corresponding 2‐month‐old mice.

**Figure S4:** Effect of 1, 5 and 10 mg.kg^−1^ soluble activin type IIB receptor delivery on skeletal muscle mass in KI mice. (a) Schematic view of the dose–response experimental design. KI mice were treated twice a week by intraperitoneal injections of soluble activin type IIB receptor (sActRIIB‐Fc) at dosage of 1, 5 and 10 mg.kg^−1^ starting at 8 weeks for 4 consecutive weeks. Tissues were removed at the end of the treatment (12 weeks of age). (b) Body weight of WT‐PBS mice (*n* = 5), KI‐PBS mice (*n* = 5), KI‐1 mg.kg^−1^ mice (*n* = 5), KI‐5 mg.kg^−1^ mice (*n* = 4) and KI‐10 mg.kg^−1^ mice (*n* = 4). (c) Relative difference of *tibialis anterior*, *quadriceps* and *gastrocnemius* muscle weight of KI‐PBS mice (*n* = 5), KI‐1 mg.kg^−1^ mice (*n* = 5), KI‐5 mg.kg^−1^ mice (*n* = 4) and KI‐10 mg.kg^−1^ mice (*n* = 4) compared to WT‐PBS mice (*n* = 5). Data are means ± SD (b) Data were analysed with a two‐way ANOVA followed by Sidak's multiple comparison test. (c) Data were analysed with a one‐way ANOVA followed by Tukey's multiple comparison test. *p < 0.05, **p < 0.01, ***p < 0.001 and ****p < 0.0001. #, significantly different from age‐matched WT‐PBS and KI‐PBS mice.

**Figure S5:** (related to Figure 5). Spleen weight, tibia length and skeletal muscle mass. (a) Spleen weight in WT‐PBS (*n* = 8), KI‐PBS (*n* = 7–13) and KI‐5 mg.kg^−1^ (*n* = 8) mice at 8 and 13 weeks of age. (b) Tibia length in WT‐PBS (*n* = 8–11), KI‐PBS (*n* = 8–13) and KI‐5 mg.kg^−1^ (*n* = 8) mice at 8 and 13 weeks of age. (c) *Quadriceps* and (d) *gastrocnemius* muscle weights in WT‐PBS (*n* = 6–8), KI‐PBS (*n* = 8) and KI‐5 mg.kg^−1^ (*n* = 11–13) mice at 8 and 13 weeks of age. Data are means ± SD (a, b, c and d) Data were analysed with a two‐way ANOVA followed by Tukey's multiple comparison test. *p < 0.05 and ****p < 0.0001. a, significantly different from corresponding 8‐week‐old mice.
