# Supplementary material for: Genetic and Pharmacologic Inhibition of Myostatin Restores Muscle Mass in a Dynamin 2 ‐Related Centronuclear Myopathy Mouse Model
Source: J Cachexia Sarcopenia Muscle. 2026 Sep 11;17(5):e70338. doi: 10.1002/jcsm.70338 (PMC13563501; doi:10.1002/jcsm.70338)

Supplemental 1

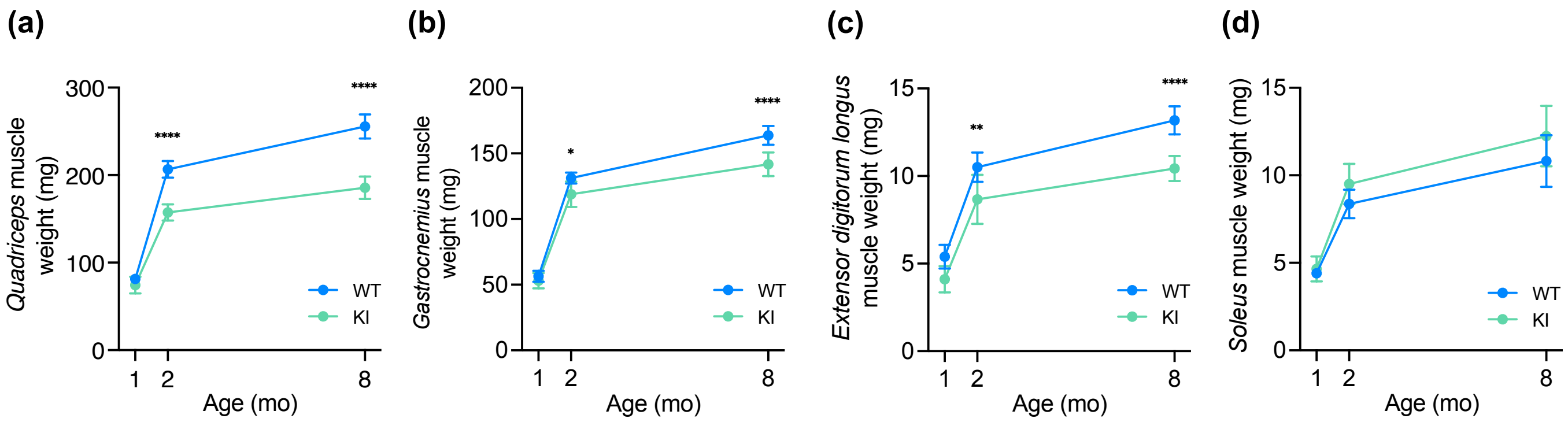

Supplement: Supplementary file 1 [file JCSM-17-e70338-s001.pdf]
