# Supplementary material for: Genetic and Pharmacologic Inhibition of Myostatin Restores Muscle Mass in a Dynamin 2 ‐Related Centronuclear Myopathy Mouse Model
Source: J Cachexia Sarcopenia Muscle. 2026 Sep 11;17(5):e70338. doi: 10.1002/jcsm.70338 (PMC13563501; doi:10.1002/jcsm.70338)
Supplement: Supplementary file 6 [file JCSM-17-e70338-s006.pdf]

Table S1. List of primers used for qPCR analysis.

Genes	Sequence accession number	Sequence (5'-3')	Length (bp) of PCR product
18S	NM_003278.3	Fwd: CGC CGC TAG AGG TGA AAT C<br>Rev: CCA GTC GGC ATC GTT TAT GG	108
45S	NR_046233.2	Fwd: GATTTCCCCGAGGTGTCGTT<br>Rev: AGAAAAGAGCGGAGGTTCGG	134
Eif4ebp1	NM_007918.4	Fwd: GGGGACTACAGCACCACTC<br>Rev: CTCATCGCTGGTAGGGCTA	171
Actb	NM_007393.3	Fwd: AGCAAGCAGGAGTACGATGAG<br>Rev: AACGCAGCTCAGTAACAGTC	80
Actr2	NM_146243.2	Fwd: TGGTGATGAGGCAAGTGAGC<br>Rev: CCCACAGGTGCTTCATGTCA	98
Actr3	NM_001205386.1	Fwd: GGAGGAAGATGGCGGGACG<br>Rev: GGGCTTGGTCACCCACTTTTG	150
Atg5	NM_053069.5	Fwd: TGAAAGAGTGTGTCCTCCTC<br>Rev: GCCTCCACTGAACTTGACTG	91
Atp1a2	NM_178405.3	Fwd: CCACCACTGCGGAAAATGG<br>Rev: GCCCTTAGACAGATCCACTTGG	143
Atp2a1	NM_007504.2	Fwd: TGTTTGTCCTATTTCGGGGTG<br>Rev: AATCCGCACAAGCAGGTCTTC	159
Atp2a2	NM_001110140.3	Fwd: GAGAACGCTCACACAAAGACC<br>Rev: CAATTCGTTGGAGCCCCAT	120
Bnip3	NM_009760.4	Fwd: AGATTGGATATGGGATTGGTCAAG<br>Rev: CCCTTTCTTCATAACGCTTGTG	121
Cacna1S	NM_001081023.3	Fwd: TCAGCATCGTGGAATGGAAAC<br>Rev: GTTCAGAGTGTTGTTGTCATCCT	116
Casq1	NM_009813.2	Fwd: TACAAAGCCTACGAGGACGC<br>Rev: TGCTGTCGAAGGTAGCGAAG	70
Chrna1	NM_007389.5	Fwd: ACCTGGACCTATGACGGCTCT<br>Rev: AGTTACTCAGGTCGGGCTGGT	67
Ctsb	NM_007798.3	Fwd: GAAGAAGCTGTGTGGCACTG<br>Rev: GTTCGGTCAGAAATGGCTTC	198
Ctsl	NM_009984.3	Fwd: GTGGACTGTTCTCACGCTCAAG<br>Rev: TCCGTCCTTCGCTTCATAGG	129
Foxo3	NM_019740.2	Fwd: GGAAATGGGCAAAGCAGA<br>Rev: AAACGGATCACTGTCCACTTG	94
Fbxo30	NM_001168297.1	Fwd: TCGTGGAATGGTAATCTTGC<br>Rev: CCTCCCGTTTCTCTATCACG	191
Fbxo32	NM_026346.2	Fwd: GTTTTCAGCAGGCCAAGAAG<br>Rev: TTGCCAGAGAACACGCTATG	115
Gabarapl1	NM_001168297.1	Fwd: TCGTGGAATGGTAATCTTGC<br>Rev: CCTCCCGTTTCTCTATCACG	191
Hprt1	NM_013556.2	Fwd: CAGGCCAGACTTTGTTGGAT<br>Rev: TTGCGCTCATCTTAGGCTTT	147
Map1lc3b	NM_026160.4	Fwd: CACTGCTCTGTCTTGTGTAGGTTG<br>Rev: TCGTTGTGCCTTTATTAGTGCATC	170
Mstn	NM_010834.3	Fwd: TTGGGCTTGACTGCGATGAG<br>Rev: GGCTTCAAAATCGACCGTGAG	74
Myh1	NM_030679.2	Fwd: GCGAATCGAGGCTCAGAACAA<br>Rev: GTAGTTCCGCCTTCGGTCTTG	138

Myh2	NM_001039545.2	Fwd: AGTCCCAGGTCAACAAGCTG<br>Rev: GCATGACCAAAGGTTTCACA	130
Myh4	NM_010855.3	Fwd: AGTCCCAGGTCAACAAGCTG<br>Rev: TTTCTCCTGTCACCTCTCAACA	100
Myod	NM_010866.2	Fwd: CATTCCAACCCACAGAACCT<br>Rev: CAAGCCCTGAGAGTCGTCTT	80
Rps19	NM_001360118.2	Fwd: GGAGGGTTAGATGCCCGGA<br>Rev: CACCCATTCGGGGACTTTCA	112
Rps21	NM_001355518.1	Fwd: CTGGACAAGTGAGCAGGTAGGC<br>Rev: ACAGGTCCACAAACTCGCC	172
Rpl23	NM_022891.3	Fwd: AAAGGCGATCTTTAGGGGCT<br>Rev: TGGGCATTGAGACGTGAACC	77
Rpl32	NM_172086.2	Fwd: AATTAAGCGAAACTGGCGGA<br>Rev: GATCTGGCCCTTGAACCTTCTC	70
Ryr1	NM_009109.2	Fwd: TATGCCCTCTACCCACTGCT<br>Rev: CTGTACGTCTCCTGCCTTGG	240
Sqstm1	NM_001290769.1	Fwd: AGGATGGGGACTTGGTTGC<br>**Rev:** TCACAGATCACATTGGGGTGC	178
Trim63	NM_001039048.2	Fwd: ACCTGCTGGTGGAAAACATC<br>Rev: AGGAGCAAGTAGGCACCTCA	147
Tuba1	NM_011653.2	Fwd: TGAGGAGGTTGGTGTGGATTC<br>Rev: AAACATCCCTGTGGAAGCAG	99
Ulk1	NM_009469.3	Fwd: TTCCTGTCAGTCTGGCTCCT<br>Rev: TGAACAGAGCCGTGACAAAG	137
Wasl	NM_028459.2	Fwd: GGGATGCGCTTTTAGACCAG<br>Rev: AATTCCTGAAGTGGGTGCGG	101

18S: 18S ribosomal RNA; *45S*: 45S ribosomal RNA; Eif4ebp1: Eukaryotic translation initiation factor 4E-binding protein; *Acta1*: Actin alpha; Actb: Actin beta; Actr2: Actin-related protein 2 (Arp2); *Actr3*: Actin-related protein 3 (Arp3); Aqp4: Aquaporin 4; *Atg5*: Autophagy related 5; *Atp1a2*: Sodium/potassium-transporting subunit alpha-2; *Atp2a1* and *2*: Sarcoplasmic/endoplasmic reticulum calcium ATPase 1 and 2 (SERCA1 and SERCA2); *Bnip3*: BCL2/adenovirus E1B 19 kDa protein-interacting protein 3; *Cacna1S*: Voltage-gated L-type calcium channel subunit alpha-1S; *CASQ1*: Calsequestrin-1; *Chrna1*: Acetylcholine receptor subunit alpha-1; *Ctsb*: Cathepsin B; *Ctsl*: Cathepsin L; *Foxo3*: Forkhead box protein O3; *Fbxo30*: F-box protein 30 (Musa1); Fbxo32: F-box only protein 32 (MAFbx); *Gabarapl1*: GABA type A receptor associated protein like 1; *Hprt1*: Hypoxanthine phosphoribosyltransferase 1; Map1lc3b: Microtubule associated protein 1 light chain 3 beta; *Mstn*: myostatin; *Myh1*, *Myh2,* and *Myh7*: Myosin heavy chain 1, 2 and 7 (MHC2A, MHC2X and MHC1, respectively); *Myod1*: Myogenic differentiation 1; *Rps19:* Small ribosomal subunit protein S19; *Rps21:* Small ribosomal subunit protein S21; *Rpl23:* Large ribosomal subunit protein S23; *Rpl32:* Large ribosomal subunit protein S32; *Ryr1*: Ryanodine receptor 1; *Sqstm1*: Sequestosome 1; *Trim63*: Tripartite motif containing 63 (MuRF1); *Tuba1*: Tubulin alpha 1; *Ulk1*: Unc-51 like autophagy activating kinase 1; *Wasl*: WASP like actin nucleation promoting factor.
